# Steatotic Liver Disease: Metabolic Dysfunction, Alcohol, or Both?

**DOI:** 10.3390/biomedicines11082108

**Published:** 2023-07-26

**Authors:** Katharina Staufer, Rudolf E. Stauber

**Affiliations:** 1 Division of Transplantation, Department of General Surgery, Medical University of Vienna, 1090 Vienna, Austria; 2 Division of Gastroenterology and Hepatology, Department of Internal Medicine, Medical University of Graz, 8010 Graz, Austria; rudolf.stauber@medunigraz.at

**Keywords:** non-alcoholic fatty liver disease (NAFLD), metabolic-dysfunction associated steatotic liver disease (MASLD), metabolic dysfunction-associated steatohepatitis (MASH), alcohol-related liver disease (ALD), steatotic liver disease (SLD)

## Abstract

Non-alcoholic fatty liver disease (NAFLD) and alcohol-related liver disease (ALD), both of them accounting for fatty liver disease (FLD), are among the most common chronic liver diseases globally, contributing to substantial public health burden. Both NAFLD and ALD share a similar picture of clinical presentation yet may have differences in prognosis and treatment, which renders early and accurate diagnosis difficult but necessary. While NAFLD is the fastest increasing chronic liver disease, the prevalence of ALD has seemingly remained stable in recent years. Lately, the term steatotic liver disease (SLD) has been introduced, replacing FLD to reduce stigma. SLD represents an overarching term to primarily comprise metabolic dysfunction-associated steatotic liver disease (MASLD), formerly known as non-alcoholic fatty liver disease (NAFLD), as well as alcohol-related liver disease (ALD), and MetALD, defined as a continuum across which the contribution of MASLD and ALD varies. The present review discusses current knowledge on common denominators of NAFLD/MASLD and ALD in order to highlight clinical and research needs to improve our understanding of SLD.

## 1. Introduction

Non-alcoholic fatty liver disease (NAFLD) is the most common chronic liver disease worldwide and shows a continuously increasing global prevalence of 38% in recent years, driven by the obesity pandemic [1,2]. In contrast, alcohol-related liver disease (ALD) has shown a predominantly stable prevalence in recent years, yet alcohol consumption as well as hospitalization and mortality rates attributable to ALD increased during the COVID-19 pandemic in several countries [3].

NAFLD is closely linked to obesity and the metabolic syndrome and represents the most rapidly increasing cause for chronic liver failure, hepatocellular carcinoma (HCC), and the need for liver transplantation (LT) [4]. While the impact of alcohol intake on the development and progression of NAFLD requires further clarification, excessive alcohol consumption of >60 g of ethanol (EtOH) per day for at least two weeks causes hepatic steatosis in 90% of individuals [5].

The nomenclature of NAFLD has been a matter of debate in recent years. It is perceived that the term NAFLD may have contributed to the trivialization of the disease, prolonging patient journeys until adequate treatment was offered [6]. Additionally, NAFLD associated with obesity might be regarded as self-inflicted disease neglecting non-lifestyle associated risk factors and supporting further stigmatization. This holds true as well for ALD, which had been renamed from alcoholic to alcohol-related or alcohol-associated liver disease in the past in order to destigmatize the nomenclature [5,7]. 

Thus, joint efforts of international scientific societies and patient associations have been made to rename and redefine NAFLD to destigmatize patients, improve diagnostic accuracy, and raise awareness [6]. Supporting this process, the term metabolic dysfunction-associated fatty liver disease (MAFLD) has been proposed in 2020 by an expert consensus panel based on subsuming detailed diagnostic criteria, yet independent of the amount of alcohol intake [8,9,10,11]. This led to the concern that alcohol consumption might be disregarded as an important factor in pathogenesis and liver-related morbidity and mortality, and patients’ access to optimal treatment might be even more hindered [12]. Recently, a consensus statement on new fatty liver disease (FLD) nomenclature has been published, following a Delphi process led by three large pan-national liver associations (AASLD, EASL, and ALEH), including patient advocates, industry representatives, regulatory agencies, as well as an independent external expert committee, which made the final recommendation [13]. This multinational consensus introduces the term steatotic liver disease (SLD) replacing FLD to serve as an umbrella term comprising the various etiologies of steatosis (Table 1). NAFLD is renamed to metabolic dysfunction-associated steatotic liver disease (MASLD), and its definition has been changed to include the presence of at least one of five cardiometabolic risk factors (Table 2). The new term MetALD was selected to include patients with MASLD who consume >140 to 350 g (females) and >210 to 420 g (males) EtOH per week, respectively (Table 2). This new consensus, in contrast to the concept of MAFLD, acknowledges that alcohol is an important contributor to disease pathogenesis and that substantial overlaps of NAFLD/MASLD and ALD exist, and concedes that the newly defined term MetALD comprises a patient cohort that warrants additional research. Data showing the weighted impact of distinct cardiometabolic risk factors as well as alcohol on the development of liver steatosis, fibrosis, cirrhosis, and hepatocellular carcinoma (HCC) are currently lacking.

Since progression of hepatic steatosis to cirrhosis and HCC is an important feature of both NAFLD and ALD, it is relevant to identify potentially harmful alcohol consumption and metabolic risk factors for FLD early in order to offer adequate treatment.

This review summarizes current knowledge on common denominators and specific differences between NAFLD/MASLD and ALD in clinical presentation, pathogenesis, diagnosis, and treatment in order to uncover hitherto unresearched fields and catalyze our understanding of unmet medical need.

Throughout this review article, the previous nomenclature will be used where appropriate to allow for an accurate reflection of the published literature.

## 2. Epidemiology

### 2.1. Epidemiology of NAFLD

NAFLD is the most rapidly growing indication for liver transplantation in the US and has become the most common indication for liver transplantation in the US elderly population [4,14].

According to a recent systematic review, global NAFLD prevalence in the general population increased by ≈50% from 25% in the time period of 1990–2006 to 38% in 2016–2019. Its prevalence has been estimated at 31% in North America and Australasia, 44% in Latin America, 25% in Western Europe, 37% in the Middle East/North Africa, 34% in South Asia, 33% in Southeast Asia, and 28% in Asia-Pacific countries [1,15].

The prevalence of NAFLD is rising in parallel with the global obesity epidemic. Among metabolic risk groups, prevalence of NAFLD is even higher, with >70% in patients with type 2 diabetes mellitus (T2DM) and 90% in patients with severe and morbid obesity undergoing bariatric surgery [16,17]. 

Currently, about 13% of children and adolescents are affected by NAFLD [18]. The sharp rise in childhood obesity resulting in pediatric NAFLD is especially worrisome as it is likely to translate into an even higher prevalence in adulthood [19].

### 2.2. Epidemiology of ALD

ALD accounts for 5.1% of all disease and injury globally, 5.3% of deaths are attributed to alcohol consumption exceeding those caused by diabetes, and the main cause of death among men aged 25–45 years is alcohol consumption [20]. In contrast to NAFLD, the prevalence of ALD has remained largely stable between the years of 2001 through 2016 in the US, accounting for 8% in 2015 to 2016 [21]. However, the same study reported an increase in the prevalence of patients with ALD and stage ≥3 fibrosis from 2.2% (2001–2002) to 6.6% (2015–2016). During the COVID-19 pandemic, alcohol consumption as well as ALD-associated hospitalization and mortality rates attributed to alcohol-related liver cirrhosis have been increasing in several countries, particularly in the US, Canada, and Latin America (summarized in [3]). Subsequently it is expected that the prevalence of ALD, including more severe disease stages, will increase in the near future.

### 2.3. Challenges

Currently available data on NAFLD and ALD epidemiology need to be interpreted with caution given that underlying databases largely did not account for systematic testing for alcohol consumption or systematic screening for alcohol use disorder (AUD) in NAFLD patients. Thus, the continuous increase in NAFLD prevalence might be biased by undetected harmful alcohol consumption [22].

## 3. Clinical Presentation

### 3.1. Clinical Presentation of NAFLD

Within NAFLD/MASLD, two stages are discerned: (i) non-alcoholic fatty liver (NAFL) or metabolic dysfunction-associated steatotic liver (MAFL) which shows low liver-related morbidity, and (ii) non-alcoholic steatohepatitis (NASH) or metabolic dysfunction-associated steatohepatitis (MASH) which has a higher risk for progressive hepatic fibrosis and shows substantial liver-related mortality [23]. Importantly, both stages are associated with an increased risk for cardiovascular events and non-hepatic malignancies, such as colorectal cancer [24]. In patients with NAFLD-related cirrhosis, though, hepatic complications are the leading causes of death [25].

As in other etiologies of chronic liver disease, fibrosis stage emerged as a major determinant of prognosis. Angulo et al. identified the superior prognostic value of fibrosis stage as compared to presence or absence of NASH on liver histology [26]. This finding was confirmed in a Swedish study of 223 patients with NASH followed for up to 33 years [27]. Thus, detection of advanced fibrosis in NAFLD is crucial for the estimation of hepatic risk and treatment need in general, and has become an important criterion to determine eligibility of patients for clinical trials with novel anti-inflammatory/antifibrotic drugs focusing on patients with fibrosis stage F ≥ 3 and NASH. Furthermore, a diagnosis of advanced fibrosis/cirrhosis establishes the need for HCC surveillance and screening for esophageal varices.

On the other hand, prognosis is also limited by extrahepatic factors, such as cardiovascular risk and non-hepatic malignancies. A diagnosis of NAFLD should, therefore, prompt screening for cardiovascular disease, e.g., by exercise testing or cerebrovascular sonography. It should be noted that the presence of NAFLD may indicate the cardiovascular manifestations of the underlying metabolic syndrome. Recently, in a retrospective study on 460 patients with stroke, we could show an association of mortality with increased FIB-4 values on admission [28].

### 3.2. Clinical Presentation of ALD

Similar to NAFLD/MASLD, ALD encompasses a spectrum of simple hepatic steatosis, steatohepatitis, fibrosis, cirrhosis, and HCC. Additionally, patients may suffer from severe alcoholic steatohepatitis (ASH) that may present as acute-on-chronic liver failure associated with high liver-related short-term mortality [29]. Notably, burden from alcohol-related liver cirrhosis is directly associated with the amount of alcohol consumed [30]. Following excessive alcohol consumption of >60 g EtOH/day for at least 2 weeks, 90% of patients present with liver steatosis, of whom 20% to 40% may develop fibrosis, and 10% to 35% may develop steatohepatitis [5].

**Figure 1:**
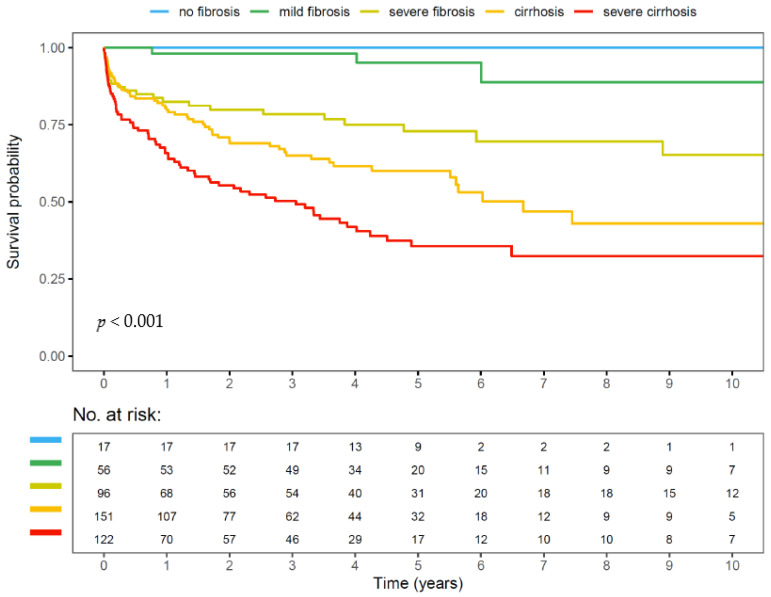
Survival of patients with ALD by SALVE fibrosis stage (SFS) (adapted from [31]).

Besides the well-known impact of abstinence during follow-up, prognosis of ALD, similarly to NAFLD, is mainly determined by fibrosis stage. In a large multinational cohort of 450 patients with ALD of varying severity, SALVE fibrosis stage (SFS) at baseline was found to be an excellent predictor of outcome after 10 years (Figure 1) [31].

However, alcohol consumption not only increases liver-related morbidity and mortality but affects numerous extrahepatic organs. Similar to NAFLD, diabetes, cardiovascular disease, and obesity are highly prevalent in patients with ALD. According to a recent meta-analysis by Theodoreson et al., major causes of extrahepatic morbidity and mortality comprise cardiovascular disease, non-hepatic malignancies, as well as infection associated with increased risk of death, with a relative risk of 2.4, 2.2, and 8.2, respectively [32]. 

### 3.3. Challenges

NAFLD/MASLD and ALD share a common picture of clinical presentation, have large overlaps in associated comorbidities, such as cardiovascular disease, obesity, and cancer, and also share fibrosis as determinant of prognosis. However, longitudinal long-term studies on the natural history of NAFLD/MASLD and ALD are needed to better understand the dynamic of both diseases as well as to facilitate public health initiatives to improve prevention and therapy. Recently, using a Delphi process, a multidisciplinary panel defined a global research priority agenda to enhance public health responses to SLD [33]. This panel highlighted, amongst others, the need to conduct “cohort studies to prospectively monitor outcomes in patients with defined liver disease phenotypes (e.g., NASH, NASH with fibrosis, cirrhosis, hepatocellular carcinoma)”, as well as to “develop and validate risk prediction models to forecast progressive hepatic and extrahepatic outcomes, to inform clinical decision making”.

## 4. Pathogenesis

### 4.1. Pathogenesis of NAFLD

NAFLD pathogenesis is multifactorial and seems to be mainly driven by lipotoxicity, insulin resistance, and inflammatory pathways. It remains unclear why only 20% of NAFLD patients progress to NASH [34].

Overload of free fatty acids leads to increased fat accumulation from de-novo lipogenesis as well as impaired lipolysis, resulting in lipotoxicity. The latter leads to hepatocellular injury, which provokes inflammation and activation of hepatic stellate cells to myofibroblasts, resulting in enhanced fibrogenesis.

Multiple hits stressing the liver, including gut-derived pathogen-associated molecular patterns (PAMPs) and or damage-associated molecular patterns (DAMPs) enhance the transition of NAFL to NASH [34].

In addition, genetic factors, such as patatin-like phospholipase domain-containing protein 3 (PNPLA3) variants, but also altered gut microbiota (dysbiosis), may contribute to the progression to NASH. Interestingly, a recently proposed hypothesis suggests that saccharomyces and candida species contained in the gut mycobiome are an important source of endogenous EtOH generation from ingested fructose (“autobrewery syndrome”) [35]. Additionally, fasting EtOH levels were significantly higher in children with NAFLD and positively associated with measures of insulin resistance, which may result from insulin-dependent impairments of alcohol dehydrogenase (ADH) activity in liver tissue rather than from increased endogenous EtOH synthesis [36].

**Figure 2:**
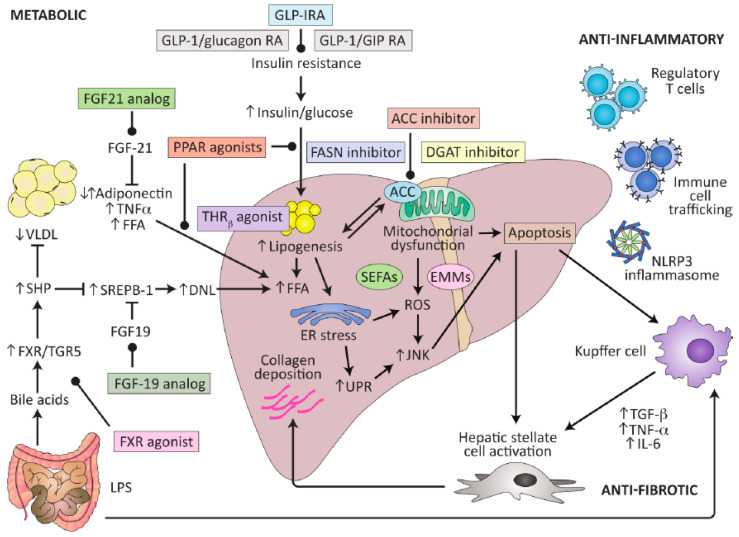
Potential therapeutic targets for NASH (from [37]; with copyright permission).

Based on current knowledge of pathogenetic factors, potential therapeutic targets have been identified, such as a reduction in fat accumulation, improvements in insulin signaling, suppression of inflammatory response, a reduction in oxidative stress, and the inhibition of fibrogenic signaling (Figure 2) [37].

### 4.2. Pathogenesis of ALD

Development of ALD and associated mortality is largely dependent on the amount of alcohol consumed. As shown by a meta-analysis by Rehm et al., >2 standard drinks per day for women and >3 standard drinks per day for men is associated with a significantly increased risk for liver cirrhosis morbidity [38]. In a more recent individual participant data analysis by Wood et al., a threshold of 100 g EtOH/week was associated with all-cause mortality in high-income countries [39].

Alcohol consumption leads to cell injury via direct and indirect toxic effects of EtOH and its even more toxic metabolite acetaldehyde. EtOH is metabolized to acetaldehyde predominantly via ADH (oxidative pathway), which is mainly expressed in the cytosol of hepatocytes (but can also be found in gastric mucosal cells, as well as lungs and kidneys) [40]. Via the GI mucosa, EtOH and its metabolites rapidly transit into the portal system and the liver. Subsequently, >80% of ingested EtOH undergoes oxidation in hepatocytes. Acetaldehyde covalently binds to proteins, phospholipids, and nucleic acids (adduct formation) leading to structural and functional protein alterations and immune responses [41,42,43,44,45].

During chronic heavy drinking, the continuous reduction of NAD+ to NADH in the reactions catalyzed by ADH and acetaldehyde dehydrogenase (ALDH) during EtOH and acetaldehyde metabolism causes a significant metabolic shift towards fatty acid synthesis as well as increased production of lactate causing metabolic acidosis (REF). Structural mitochondrial alterations caused by acetaldehyde lead to decreased ATP generation and the production of reactive oxygen species causing oxidative stress (reactive oxygen species, ROS, such as H_2_O_2_ and O^2^^−^), DNA damage or lipid peroxidation of cell membranes, and increased endotoxin generation and inflammation. 

Alcohol is additionally metabolized via cytochrome P450 2E1 (CYP2E1) (non-oxidative pathway or alternative pathway), which can be induced by increasing amounts of alcohol as EtOH protects CYP2E1 from being degraded by the ubiquitin proteasome system [46]. This leads to subjectively increased alcohol tolerability, but also increased metabolization to acetaldehyde and increased production of ROS. 

EtOH and acetaldehyde also show direct toxic effects onto the gastric and intestinal mucosa which may lead to maldigestion and malabsorption of nutrients, vitamins, and trace elements. In the small and large intestine, EtOH is metabolized by the residing microbiota leading to an additional increase in acetaldehyde, cellular damage, and dysbiosis, including changes in the mycobiome and virome (mainly bacteriophages) [47]. 

Despite the toxic effects of alcohol, only up to 20% of patients with continued excessive alcohol consumption develop alcohol-related liver cirrhosis, pointing to additional protective and risk factors in ALD pathogenesis. As well as the amount of alcohol consumed, the time period of continued alcohol consumption and the drinking pattern are important contributors: daily alcohol consumption, drinking while being fasted and binge drinking have been identified as determinants of increased risk [7,9]. 

Although conflicting data exist, the type of alcohol may also be associated with the risk of ALD development. While beer and red wine have been found to be associated with less harm to the liver in animal models and human studies, others did not confirm these findings [30,48,49,50].

Additionally, genetic ancestry and sex modify the risk and severity of ALD and may predispose individuals to AUD [51,52]. Patients of Hispanic ethnicity may present with ALD at earlier ages than patients of Caucasian ancestry [53]. Although men are more frequently affected by AUD, ALD, and consume higher amounts of alcohol, women show higher susceptibility to EtOH-induced liver toxicity [54].

Similar to NAFLD, several single nucleotide polymorphisms (SNP) have also been identified as modifiers of the risk and severity of ALD [55,56]. Based on a large genome-wide association study, PNPLA3, transmembrane 6 superfamily member 2 (TM6SF2), and membrane-bound O-acyltransferase domain-containing protein 7 (MBOAT7) were determined to be associated with ALD progression presumably via modulating lipid metabolism and/or HCC risk, for which the exact mechanisms have remained unclear [57]. Further studies have identified an association between susceptibility to ALD and polymorphisms in the pattern recognition receptor CD14 gene, as well as in the nuclear factor erythroid 2-related factor 2 gene, both of which are involved in endotoxin-mediated inflammation and susceptibility to ALD [58,59]. Larger studies are needed to confirm these findings. 

Furthermore, cultural influences, environmental factors, and diet are factors contributing to the development and severity of ALD [53,54,60,61]. 

### 4.3. Challenges

As well as epidemiology and clinical presentation, NAFLD/MASLD and ALD have several pathogenetic determinants in common. However, pathogenesis of NAFLD and ALD is still incompletely understood. Elucidating the interaction of EtOH and food intake/weight, as well as the role of microbiota, including the intestinal barrier, might help identify biomarkers for early diagnosis and new therapeutic targets. 

## 5. Diagnosis

### 5.1. Noninvasive Risk Stratification in NAFLD

Due to the high prevalence of NAFLD risk stratification by simple noninvasive tests, it is of utmost importance to provide optimal linkage to care for those patients with high risk of liver-related events. Previous studies have identified advanced fibrosis (F ≥ 3) as the major prognostic factor in NAFLD [26,27,62]. The presence of NASH and advanced fibrosis on liver histology is currently the primary outcome parameter in clinical trials evaluating anti-inflammatory and/or antifibrotic pharmacologic therapy. However, due to the high prevalence of NAFLD, universal liver biopsy is not feasible and noninvasive triage tests are needed either as an alternative or to select patients for liver biopsy [63,64,65].

While there is currently no approved biomarker for the presence of NASH, several noninvasive tests are available for the estimation of fibrosis stage in NAFLD. Simple tests based on routine clinical and laboratory parameters are readily available and are, therefore, useful for broad risk stratification in primary care. 

NAFLD fibrosis score (NFS), using a combination of the parameters of age, fasting glucose, body mass index (BMI), platelet count, albumin, and AST/ALT ratio [66], as well as fibrosis-4 index (FIB-4), including age, AST, ALT, and platelet count [67], are among the most widely used noninvasive tests. On direct comparison, FIB-4 shows equal or slightly superior diagnostic accuracy for the detection of advanced fibrosis and seems preferable as its calculation may be easily implemented in clinical laboratories.

In addition, several proprietary liver fibrosis panels have been developed. The enhanced liver fibrosis (ELF™) test is based on three extracellular matrix proteins including hyaluronic acid (HA), procollagen-3 N-terminal peptide (P3NP), and tissue inhibitor of metalloproteinase-1 (TIMP-1), and has been studied in adult and pediatric patient populations with NAFLD [68,69,70,71]. However, proposed cut-offs vary between 9.8 and 10.5 for the diagnosis of advanced fibrosis [72,73,74]. Other fibrosis panels combining routine and proprietary tests include FibroMeterV2G (platelet count, prothrombin index, AST, alpha-2-macroglobulin, hyaluronic acid, urea, age, and sex) [75,76], FibroMeterV3G (using the same parameters as FibroMeterV2G, yet GGT instead of hyaluronic acid) [77], and FibroTest (alpha-2-macroglobulin, haptoglobin, GGT, age, bilirubin, apolipoprotein A1, and sex) [78]. While these panels perform slightly better than the above-mentioned simple tests, their determination is costly.

Vibration-controlled transient elastography (VCTE, FibroScan^®^) has shown accurate fibrosis staging ability in liver diseases of various etiologies including NAFLD [79,80,81,82] and allows for simultaneous estimation of hepatic fat content using the continuous attenuation parameter (CAP) feature. The measurement of liver stiffness (LSM) provides superior diagnostic accuracy for advanced fibrosis; however, this method is limited to referral centers due to the high equipment cost and has a substantial failure rate, especially in obese patients [83,84,85].

Recent studies have focused on the sequential use of a simple test (mostly FIB-4) followed by a detailed noninvasive staging by the ELF test or VCTE. These sequential strategies are cost-effective as the high NPV of FIB-4 < 1.30 for advanced fibrosis allows researchers to reserve the costlier proprietary tests for those patients above this cut-off. In a recent individual patient data meta-analysis on 5735 patients from 37 studies, Mozes et al. validated the FIB-4 followed by VCTE algorithm, indicating high NPV for FIB-4 < 1.30 and reasonable PPV for FIB-4 > 2.67 or LSM > 10 kPa [86]. Importantly, analysis of follow-up data in the same cohort revealed that the presence of advanced fibrosis assessed noninvasively was an excellent predictor of later development of both liver-related and cardiovascular events [87].

### 5.2. Noninvasive Risk Stratification in ALD

Unfortunately, ALD is mostly detected in very advanced stages presenting with decompensated cirrhosis. Nevertheless, noninvasive fibrosis tests may be well-suited in screening for early ALD, e.g., during or shortly after alcohol detoxification treatment.

Thiele et al. evaluated and compared 10 different noninvasive fibrosis tests in a Danish cohort of 289 patients with ALD attending an outpatient liver clinic or rehabilitation and demonstrated good to excellent diagnostic accuracy for the prediction of F3-4 stage on histology using FIB-4 (AUROC 0.85), ELF score (AUROC 0.92), and VCTE by FibroScan (AUROC 0.89 by intention-to-diagnose, 0.97 per protocol) [2]. 

It should be noted that liver stiffness measured by VCTE depends not only on fibrosis but also inflammation, cholestasis, and/or congestion. During alcohol withdrawal, a rapid decline in LSM has been observed within 2 weeks after admission [88]. In order to avoid false high LSM values in the presence of steatohepatitis, a correction factor according to AST level has been proposed [88]. 

An individual patient data meta-analysis of 10 studies comprising the VCTE data of 1026 patients with ALD revealed an optimal LSM cut-off of 12.1 kPa for the prediction of F ≥ 3, which, however, varied from 8.8 kPa to 16.1 kPa depending on AST and bilirubin levels [89].

### 5.3. Liver Histology in NAFLD

Histological NASH is defined by the presence of >5% macrovesicular steatosis, lobular inflammation, and hepatocellular ballooning, typically with a predominantly centrilobular distribution [90]. Activity may be summarized by the NAFLD Activity Score (NAS) ranging from 0–8 [91]. Fibrosis is usually staged according to the Clinical Research Network (CRN) score [91]. Other more detailed scoring systems have been developed (Steatosis, Activity, and Fibrosis score, SAF score) but are not yet widely used in clinical practice.

### 5.4. Liver Histology in ALD

According to Yip et al., histological ASH is defined by the presence of steatosis (any degree), lobular inflammation, and hepatocellular ballooning [92]. Grading and staging has been traditionally performed using the CRN system originally described for NAFLD. However, this does not account for peculiar histologic features of ASH, such as canalicular cholestasis, ductular cholestasis, pericellular fibrosis, and thick fibrous septa. An improved classification has been recently developed by the SALVE Histopathology Group and validated in a large European cohort of 450 patients with ALD of varying severity [31].

### 5.5. Challenges

Alongside the increasing burden of obesity and T2DM, alcohol consumption has become a major public health concern given that 55.5% of the world population, 76.5% of the European population, and 83.1% of the American population consume alcohol (estimated by the World Health Organization in 2016) [20]. The average amount of pure EtOH ingested by people consuming alcohol is 32.8 g/day globally [20], associated with increased liver-related morbidity, cardiovascular disease, and all-cause mortality. Thus, early diagnosis of patients with potentially harmful alcohol consumption is needed to provide adequate treatment. None of the presented diagnostic tools are able to reliably differentiate NAFLD from ALD. In order to advance our in-depth understanding of pathogenesis, natural disease history, and to offer accurate and timely treatment, systematic screening for alcohol consumption as well as metabolic risk factors is mandatory. Application of validated questionnaires for AUD, such as the Alcohol Use Disorders Identification Test (AUDIT) questionnaire (or its short form AUDIT-Consumption, AUDIT-C) should be considered in every liver disease evaluation including at the primary care level. Questionnaire screening instruments should be complemented by measuring objective alcohol biomarkers, such as urinary ethylglucuronide (uEtG), EtG in hair (hEtG), or phosphatidylethanol (PEtH) [22,93].

## 6. Treatment

### 6.1. Treatment of NAFLD 

#### 6.1.1. Lifestyle Modification

Lifestyle modification resulting in weight loss currently represents the cornerstone of NAFLD treatment as its beneficial effects have been demonstrated in multiple studies. In a small randomized study, Promrat et al. tested the effect of intensive lifestyle intervention vs. structured education only on NASH and reported a significant improvement in ALT and a reduction in NAS on liver histology in parallel to weight loss of 9% in the intervention group [94]. These findings were later confirmed in a large study enrolling 293 patients with histologically proven NASH who adopted lifestyle changes over 52 weeks and underwent liver biopsy before and after this intervention. All patients received a hypocaloric diet recorded in a daily food diary and were encouraged to walk 200 min per week. This led to resolution of steatohepatitis in 25% and regression of fibrosis in 19%. Even better results were obtained in a subgroup who achieved >10% of weight reduction, with resolution of steatohepatitis in 90% and regression of fibrosis in 45% [95].

The Western diet, containing high amounts of saturated fat and carbohydrates, should be replaced by Mediterranean diet rich in polyphenols and omega-3 polyunsaturated fatty acids [96]. Low-fat diets, low-carb diets, and time-restricted eating have been proposed as alternative approaches.

#### 6.1.2. Drug Therapy for NASH

As the above-mentioned lifestyle changes are achieved in a minority of NAFLD patients only, effective drug therapy is highly desirable, especially in patients with active fibrotic NASH. Pharmacologic treatment of NASH aims to improve various pathophysiologic pathways, including metabolic (insulin resistance, lipotoxicity, oxidative stress), anti-inflammatory, and anti-fibrotic targets (Table 3). To date, no licensed NASH medication exists despite extensive preclinical and clinical research activities. Numerous clinical trials have been designed for treatment of precirrhotic NASH (mainly enrolling patients with fibrosis stage F2–F3, endpoint resolution of NASH, and/or improvement of fibrosis stage) and cirrhotic NASH (endpoint reduction in liver-related events). Drugs currently in phase 3 studies are listed in Table 4. Among these, resmetirom is the first drug that achieved both efficacy endpoints on liver histology, i.e., (i) resolution of NASH without worsening of fibrosis, and (ii) improvement of CRN fibrosis stage, in a recent phase 3 trial. On the other hand glucagon-like peptide-1 (GLP-1) agonists are promising candidates for NASH treatment due to their ability to induce sustained weight loss on long-term treatment, such as a 15% weight loss achieved by weekly subcutaneous semaglutide at 2.4 mg for 68 weeks [97].

#### 6.1.3. Bariatric Surgery

Bariatric surgery, using the Roux-en-Y gastric bypass or sleeve gastrectomy, is a very effective treatment option in patients with morbid obesity (BMI > 40 kg/m^2^). A French study with serial liver biopsies at the time of surgery, 1 year later, and 5 years later has demonstrated resolution of NASH without worsening of fibrosis at 5 years in 84% and improvement of fibrosis by ≥1 stage at 5 years in 70% of patients [103,104]. However, the surgical approach is limited in patients with advanced stages of cirrhosis due to high perioperative mortality.

### 6.2. Treatment of ALD

Treatment of AUD and ALD requires an integrated approach comprising medical management (addiction specialist, hepatologist), psychosocial intervention, and pharmacological therapy [105]. Although it has become a well-established treatment concept to aim at reducing and controlling the amount of alcohol intake patients with AUD, patients with ALD should always aim at alcohol abstinence in order to hold or reduce disease progression and improve prognosis.

In order to support abstinence, pharmacological treatment options in patients with ALD are limited. Baclofen is currently the only pharmacological treatment (off-label use) that has been tested in randomized-controlled trials in patients with AUD and advanced ALD, and its safety in patients with advanced liver disease has been supported by several additional observational studies [106,107,108]. Disulfiram, a drug increasing levels of acetaldehyde in blood and leading to potentially life-threatening adverse effects, is contraindicated in patients with liver cirrhosis. Substantial evidence to use acamprosate, naltrexone, nalfemene, or sodium oxybate are currently lacking so that broader application in this patient cohort cannot be recommended or may be subject to strict benefit–risk assessment (reviewed in [109]). 

Future treatment options for liver fibrosis in ALD might include rifaximin-α, which has recently been evaluated in an investigator-initiated, randomized, double-blind, placebo-controlled, single-center, phase 2 trial at Odense University Hospital in Denmark. The trial showed a lesser fibrosis increase after 18 months in the treatment arm [110]. Findings need to be confirmed in an upcoming phase 3 trial.

### 6.3. Treatment of MetALD

According to the new nomenclature and definition, patients with MetALD present with features of both MASLD and ALD, including hepatic steatosis, ≥1 cardiometabolic risk factor out of 5, plus average daily alcohol consumption of 20–50 g (females) and 30–60 g (males), respectively (Table 2). This new entity warrants further studies to better understand the ideal treatment options for MetALD. Considering that a substantial number of patients with NAFLD/MASLD may consume unreported amounts of alcohol [22,93], this may have influenced the outcome of clinical trials for the treatment of NASH in the past in such a way that the endpoints as currently accepted by regulatory authorities (to improve fibrosis without worsening of NAS, or NASH resolution without worsening of fibrosis) were not met (Table 4). Conversely, drugs working in NASH/MASH might not automatically be effective in MetALD. 

### 6.4. Challenges

It is well known that concurrent alcohol consumption worsens the prognosis of NAFLD/MASLD and ALD. However, the pathogenesis of NAFLD/MASLD and ALD is multifactorial, incompletely understood, and the true impact of alcohol consumption on the development and progression of SLD has remained unclear. Further research is needed to delineate the relative risk conferred by metabolic factors, various amounts (and type) of alcohol, and further pathomechanistic factors that might influence disease development, progression, as well as treatment targets. 

Importantly, current treatment of SLD should aim for both correction of cardiometabolic factors and alcohol abstinence. The designs of future clinical trials on the treatment of MASH, MetALD, and ALD need to consider characteristics of both MASLD and ALD.

## 7. Future Directions 

Understanding that there might be a huge overlap between NAFLD/MASLD and ALD, including the new entity of MetALD, requires further clarification of disease-contributing factors. Moving forward, a systematic screening for alcohol consumption and AUD as well as cardiometabolic risk factors is needed in patients presenting with (suspected) chronic liver disease. This should include the assessment of the amount of alcohol consumed with application of alcohol biomarkers to understand the true burden of disease, allow early diagnosis, and improve access to accurate treatment. Improving our understanding of the decisive drivers of NAFLD/MASLD and ALD, as well as their mutual interaction, will support further refinement of the MetALD definition and allow for a personalized treatment approach.

## Figures and Tables

**Table 1:** New nomenclature according to a recent multi-society * Delphi consensus statement [13].

Old Nomenclature	New Nomenclature
Fatty liver disease (FLD)	Steatotic liver disease (SLD)
Nonalcoholic fatty liver disease (NAFLD)	Metabolic dysfunction-associated steatotic liver disease (MASLD)
Nonalcoholic steatohepatitis (NASH)	Metabolic dysfunction-associated steatohepatitis (MASH)
---	MetALD: MASLD with average alcohol intake of 20–50 g/day (females)/30–60 g/day (males)
Alcohol-related liver disease (ALD)	Alcohol-associated liver disease (ALD)
---	Specific etiology SLD
---	Cryptogenic SLD

* led by AASLD, EASL and ALEH.

**Table 2:** Diagnostic criteria of fatty liver disease sub-categories: former vs. new nomenclature and definition.

Former Nomenclature			New Nomenclature	
	EASL-EASO-EASD [7,8]	AASLD [5,9]		Multi-Society Delphi Consensus 2023 [13]
NAFLD	- Excessive hepatic fat accumulation: steatosis present in >5% of hepatocytes according to histological analysis or by a proton density fat fraction > 5.6% assessed by proton magnetic resonance spectroscopy (^1^H-MRS) or quantitative fat/water selective magnetic resonance imaging (MRI). - Exclusion of both secondary causes and of a daily alcohol consumption ≥ 20 g for women and ≥ 30 g for men. - Associated with metabolic risk factors/components of metabolic syndrome: Waist circumference ≥ 94/≥ 80 cm for Europid men/women; Arterial pressure ≥ 130/85 mmHg or treated for hypertension; Fasting glucose ≥ 100 mg/dL (5.6 mmol/L) or treated for T2DM; Serum triacylglycerols > 150 mg/dL (>1.7 mmol/L); HDL cholesterol < 40/50 mg/dL for men/women (<1.0/<1.3 mmol/L).	- Evidence of hepatic steatosis (≥5%) either by imaging or histology in the absence of significant alcohol consumption (ongoing or recent alcohol consumption ≤ 21 standard drinks on average per week in men and ≤14 standard drinks on average per week in women; 1 standard drink is equivalent to 14 g EtOH). - Lack of secondary causes of hepatic fat accumulation, such as significant alcohol consumption, long-term use of a steatogenic medication, or monogenic hereditary disorders - Commonly associated with metabolic comorbidities, such as obesity, diabetes mellitus, and dyslipidemia.	MASLD	- Presence of hepatic steatosis and ≥1 cardiometabolic risk factor out of 5 (see below) and no other discernible cause for hepatic steatosis; - If additional drivers of steatosis are identified, then this is consistent with a combination etiology. - Cardiometabolic risk factors: BMI ≥ 25 kg/m^2^ (23 Asia) OR waist circumference > 94 cm (males)/> 80 cm (females) OR ethnicity-adjusted; Fasting serum glucose ≥ 5.6 mmol/L (100 mg/dL) OR 2-h post-load glucose levels ≥ 7.8 mmol/L (≥140 mg/dL) OR HbA1c ≥ 5.7% (≥39 mmol/L) OR T2DM OR treatment for T2DM; Blood pressure ≥ 130/85 mmHg OR specific antihypertensive drug treatment; Plasma triglycerides ≥ 1.70 mmol/L (≥150 mg/dL OR lipid lowering treatment; Plasma HDL cholesterol ≤ 1.0 mmol/L (≤40 mg/dL) (males) and ≤1.3 mmol/L (≤50 mg/dL) (females) OR lipid lowering treatment.
Secondary NAFLD	- e.g., AFLD, drug-induced FLD, hepatitis C virus-associated fatty liver (genotype 3), etc. Note that primary and secondary NAFLD may coexist in individual patients. Also, NAFLD and AFLD may coexist in subjects with metabolic risk factors and drinking habits above safe limits.		Specific etiology SLD	- Hepatic steatosis due to drug-induced liver injury, monogenic diseases (e.g., Wilson´s disease), and miscellaneous (e.g., Hepatitis C virus).
			Cryptogenic SLD	- Patients with steatosis without cardiometabolic risk factors or other discernible causes. - If metabolic dysfunction is strongly suspected despite the absence of cardiometabolic risk factors, the term “possible MASLD” can be considered pending additional testing.
			MetALD	- MASLD plus average alcohol intake of 20–50 g/day (females) and 30–60 g/day (males).
ALD	- Usually suspected upon documentation of regular alcohol consumption of >20 g EtOH/day (females) and >30 g EtOH/day (males) in the presence of clinical and/or biological abnormalities suggestive of liver injury.	- Spectrum of liver injury resulting from alcohol use, ranging from hepatic steatosis to alcoholic hepatitis, alcohol-associated cirrhosis, and acute alcoholic steatohepatitis in patients with >1 standard drink/24 h (females) or >2 standard drinks/24 h (males) (one standard drink is equivalent to 14 g EtOH).	ALD	- Liver injury in patients with average alcohol intake of >50 g/day (females) and >60 g/day (males).

**Table 3:** Potential targets of pharmacologic NASH treatment (studied in phase 2 and 3 trials).

Mode of Action	Drug
Farnesoid X receptor (FXR) agonist	Obeticholic acid
Peroxisome proliferator-activated receptor (PPAR) agonists	Pioglitazone Elafibranor Lanifibranor
Fibroblast growth factor 21 (FGF21) agonists	Aldafermin Efruxifermin Pegozafermin
Glucagon-like peptide-1 (GLP-1) agonist	Liraglutide Semaglutide
Dual GLP-1/glucagon agonists	Cotadutide Efinopegdutide
Sodium/glucose transport protein 2 (SGLT2) inhibitor	Empaglifocin
Thyroid hormone receptor ß (TRß) agonist	Resmetirom
CCL receptor type 2 (CCR2) and type 5 (CCR5) antagonist	Cenicriviroc
Antioxidant	Vitamin E

**Table 4:** Phase 3 trials of drug therapy in F2/F3 NASH.

Substance	Class	Acronym	Result	Ref.
Obeticholic acid	FXR agonist	REGENERATE	Fibrosis endpoint * met on interim analysis, but relevant AEs (pruritus, LDL elevation)	[98] NCT02548351
Elafibranor	PPARα/δ agonist	RESOLVE-IT	Efficacy endpoints not met at interim analysis	[99] NCT02704403
Selonsertib	ASK1 inhibitor	STELLAR-3	Efficacy endpoints not met at interim analysis	[100] NCT03053050 and NCT03053063
Cenicriviroc	CCR2/5 inhibitor	AURORA	Efficacy endpoints not met at interim analysis	[101] NCT03028740
Resmetirom	THR-ß agonist	MAESTRO-NASH	Fibrosis * and NASH ** endpoints met on interim analysis	[102] NCT03900429
Lanifibranor	Pan-PPAR agonist	NATiV3	Ongoing	NCT04849728
Semaglutide	GLP1 agonist	ESSENCE	Ongoing	NCT04822181

* improvement by ≥1 stage of fibrosis with no worsening of NAS. ** NASH resolution without worsening fibrosis.
